# Combining reconstructive and ablative surgical treatment of chronic breast cancer-related lymphedema (BCRL): safe and effective

**DOI:** 10.1007/s10549-022-06778-y

**Published:** 2022-10-26

**Authors:** Alina A. Ghazaleh, Tristan M. Handschin, Julia Buckowiecki, Frédérique S. Chammartin, Christoph Andree, Dirk J. Schaefer, Martin Haug, Elisabeth A. Kappos, Katrin Seidenstuecker

**Affiliations:** 1 Department of Plastic, Reconstructive and Aesthetic Surgery, Sana Hospital Benrath, Duesseldorf, Germany; 2 Department of Plastic, Reconstructive and Aesthetic Surgery, Sana Hospital Gerresheim, Duesselorf, Germany; 3 Department of Plastic, Reconstructive, Aesthetic and Hand Surgery, University Hospital of Basel, Basel, Switzerland; 4 University of Basel, Basel, Switzerland; 5 Basel Institute for Clinical Epidemiology and Biostatistics, Department of Clinical Research, University Hospital and University of Basel, Basel, Switzerland; 6 Breast Center, University Hospital of Basel, Basel, Switzerland

**Keywords:** Breast cancer-related lymphedema, Chronic lymphedema, Breast cancer, Lymphatic surgery, VLNT, WAL

## Abstract

**Purpose:**

We investigated whether a one-stage combination of vascularized lymph node transfer (VLNT) with water jet-assisted liposuction (WAL) can be safely performed and results in improved patient outcomes such as a greater reduction in arm volume when treating chronic breast cancer-related lymphedema (BCRL).

**Methods:**

In this retrospective cohort study, we included all patients from our encrypted lymphedema database treated for chronic BCRL with VLNT or VLNT + WAL who had a minimum follow-up of two years. We analyzed patient-specific variables including arm circumferences as well as patient-reported outcomes before and after surgery as well as surgery time, surgery-related complications and patient satisfaction.

**Results:**

Only the mean preoperative differences of the circumferences between the lymphedematous and the unaffected arm in individual patients showed a statistically significant difference between treatment groups (*p* < 0.05). Indeed, patients treated with VLNT + WAL had consistently larger differences in individual sets of arms and therefore more pronounced chronic BCRL. The mean surgery time was significantly longer in the VLNT + WAL group (*p* < 0.05). Complications were seldom and similar in both groups. Using a numeric rating scale, the level of patient satisfaction following treatment did not differ significantly between groups (*p* = 0.323).

**Conclusions:**

Our findings suggest that a one-stage combination of VLNT with WAL does not result in more complications even though it also entails a longer surgery time. This is acceptable as secondary interventions resulting in overall longer surgery times and higher costs can be avoided. A one-stage combination might be especially favourable for patients suffering from more severe chronic BCRL.

## Introduction

### Relevance of chronic lymphedema after breast cancer treatment

While breast cancer surgery has evolved, postoperative complications still occur impacting patient satisfaction and quality of life. In fact, chronic breast cancer-related lymphedema (BCRL) belongs to one of the most frequent surgery-related complications with more than one in five survivors affected [1]. This is largely due to the fact that breast cancer treatment nearly always necessitates sentinel lymph node dissection at best and complete axillary lymph node dissection at worst [2]. Nevertheless, a de-escalation of breast cancer surgery has taken place with ever fewer lymph nodes requiring dissection [3–5].

### Repercussions of chronic BCRL

Chronic BCRL takes a toll on physical and psychological well-being [1, 6]. Secondary symptoms include limited range of motion, heaviness, pain and numbness in the upper extremity, decreased grip strength as well as body dysmorphia, increased self-consciousness, sadness, anger and anxiety [7–15]. Also, chronic lymphedema has serious socioeconomic effects on both the patient and society as a whole. For example, cancer survivors with chronic BCRL cover up to 112% higher out-of-pocket costs and are subjected to detrimental effects on work and career and society is faced with high direct and indirect expenses in the form of significant healthcare expenditures and opportunity costs [16–18].

### The standard treatment of chronic lymphedema

The standard treatments for chronic BCRL are purely symptomatic. They include compression techniques, manual lymphatic drainage and exercise, among others [19]. These treatments do not address the underlying pathomechanisms as the integrity of the lymphatic system is not restored. Moreover, current treatment guidelines recommend conservative complex physical decongestion therapy (CDT)—the gold standard to date—that includes gentle massage, local compression, physical exercise and meticulous skin care [20]. Unfortunately, these conservative procedures are limited in their clinical effectiveness as Jeffs et al. demonstrated in their systemic review [21].

### Vascularized lymph node transfer (VLNT) and liposuction in chronic lymphedema

Unlike all the conservative treatments available, several microsurgical procedures now exist that can properly address the actual causes of chronic lymphedema [21].

VLNT involves the autologous microvascular relocation of lymph nodes, for instance from the groin, to a lymphedematous region [22–24]. These fully functional lymph nodes are then connected via vascular anastomosis to the recipient vessels as a free flap. This new network of lymphatic pathways within the flap then acts like a sponge absorbing excessive lymphatic fluid in the lymphedematous region and draining it into the venous system [25]. In time, lymphangiogenesis occurs as well [26]. These two main mechanisms ultimately bolster the local lymphatic drainage system [25, 26].

A systematic review of 18 studies by Ozturk et al. concluded that VLNT leads to a significant improvement of lymphedema as was expressed by multiple patient outcomes, e.g. reduction of limb volume [22]. Coriddi et al. systematically reviewed 32 studies focussing on quality of life and found significantly improved outcomes for physiologic surgical treatment of lymphedema with VLNT accounting for 11 of the examined studies [27]. Hence, VLNT is a viable treatment option for chronic lymphedema.

Liposuction aims to reduce the local tissue by removing hypertrophied adipose tissue—a hallmark of chronic lymphedema—thus relatively improving the local lymphatic drainage [28]. Liposuction is a viable and widely used treatment for lymphedema which was confirmed by two systematic reviews by Forte et al. who found improved quality of life and reduction of limb volume after treatment [29, 30].

### Study premise

Both VLNT and water jet-assisted liposuction (WAL) have shown their effectiveness in the treatment of chronic lymphedema in general and chronic BCRL in particular [22, 27, 29, 30]. A systematic review by Forte et al. on liposuction and lymph node transfer for the treatment of BCRL concluded that a combination of both techniques may lead to improved patient outcomes such as a decrease in volumes in the affected upper extremities [31]. However, of the five studies included in this systematic review, four studies had used VLNT and liposuction in two stages and did so without a control group. The one study that compared VLNT with VLNT and liposuction concurrently was undertaken by Leppäpuska et al. from 2007 to 2015 [32]. Principally, they showed for the first time that liposuction can be safely performed with VLNT during one surgery. However, Leppäpuska et al. predominantly used dry liposuction and did not statistically analyze the differences in arm volumes pre- or postoperatively between the two groups. Therefore, a one-stage combination of VLNT with WAL in chronic BCRL may not only be safe but also result in improved patient outcomes such, e.g. a greater reduction in arm volume.

For ablative liposuction, tumescent solution containing adrenaline is used which leads to vasoconstriction. There are reservations amongst surgeons about combining VLNT with WAL concurrently as vasoconstriction of the flap microcirculation might lead to increased flap complications. Nevertheless, as current literature deems both VLNT and WAL to be low in complications, a one-stage combination might not necessarily lead to more complications and retain high patient safety [22, 25, 27–30]. As such, the main objective of our study was to determine whether a one-stage combination of VLNT with WAL results in better patient outcomes than VLNT alone in the treatment of chronic BCRL without resulting in more complications. Additionally, our aim was to compare patient-specific variables including BMI and patient satisfaction.

## Methods

### Surgical procedure

Prior to surgery, we localize the vascular pedicle of the flap (superficial circumflex iliac artery/vein) and the superficial inferior epigastric vein with either a handheld acoustic Doppler probe or using duplex ultrasound in order to mark their course on the skin [33]. The lymph nodes in between these two landmarks are the ones meant for transplantation as they drain the lateral abdominal wall and not the donor site leg. Viitanen et al. described the anatomical landmarks including the danger zones when it comes the donor site morbidity [34]. The superficial epigastric vein represents the medial limitation of the flap.

Before surgery, the patient receives a single shot of antibiosis. For reverse mapping of the extremity, 0.04 cc of patent blue are injected intradermally between each toe.

For the ablative liposuction, 300–400 cc of tumescent solution are applied into the arm in a longitudinal direction. Thereafter, we perform a water assisted liposuction of the entire arm—leaving out the hand and the medial bicipital groove—with a 3.5–4.2 mm cannula. This procedure must be done strictly longitudinally to preserve the lymphatic system. After the liposuction, we suture the incisions with a single stitch and wrap the arm with sterile bandages.

In the axilla, the scar of the axillary dissection can be reused and, if needed, expanded via Z-plasty to prepare the recipient vessels. Here, we aim to excise all scar tissue leading up to the axillary vein. Subsequently, we dissect the thoracodorsal and the long thoracic nerve in order to preserve them and prepare the recipient vessels. Predominantly, we use a branch of the thoracodorsal artery and vein or the thoracodorsal vessels themselves. The axillary surgery is performed using loupe magnification.

While the first team works on the aforementioned procedure, the second team contemporaneously harvests the flap from lateral to medial on the fascia. For orientation, it is useful to dissect the superficial vein first to be sure to not include any tissue medial from this important landmark. The superficial circumflex pedicle enters subfascially and mostly cranio-laterally to the superficial vein. The superficial circumflex artery and vein have a superficial and a deep branch. It is important to harvest the flap from the superficial branch. Moreover, there are a lot of lymphatic pathways underneath the fascia that should not be harmed as it increases donor side morbidity [35]. In order to obtain a sufficient diameter of the vessels, we open the fascia and dissect further towards the femoral vessels.

As the lymphatic vessels are coloured blue after patent blue injections, we are able to identify and preserve them. If a lymph node is coloured, we don’t include it into the flap to avoid secondary lymphedema of the lower extremity. Using 3.8 loupe magnification, we clip the uncoloured lymphatic vessels at the donor site while leaving them open in the flap. It is of key importance not to use any kind of coagulation or bipolar devices for the preparation of the medial-inferior border of the flap as it may cause occlusion of the lymphatic vessels relevant for the flap. If one of these lymphatic vessels under the fascia is coloured by the reverse mapping light and we can’t preserve it, we perform a lymphaticovenous anastomosis to avoid compromising the lymphatic drainage of the leg.

When the recipient vessels are prepared and have a suitable diameter for the anastomosis (> 1 mm), we transfer the flap to the axillary region and perform a microsurgical end-to-end anastomosis with 10–0 Ethilon single stiches under the microscope. The veins are oftentimes too small for a coupler anastomosis. The flap is deepithelialized and once we see punctual bleeding of the corium and have an adequate Doppler sound signal from the vessels, we place the flap as close as possible to the axillary and suture it with 3–0 Vicryl to hold it in position.

### Study design

For this study, we used the national United Kingdom specialist service’s definition for chronic lymphedema which states that lymphedema lasting longer than three months and affecting one or more areas such as the limbs, hands, and/or upper body is regarded as chronic, regardless of the cause [36].

All patients treated for chronic BCRL at one tertiary referral center were prospectively entered into an encrypted databank along with all measured variables. We searched our database for patients who were treated for chronic BCRL with either VLNT or VLNT + WAL between Jan. 1, 2015 and Dec. 31, 2020 with a follow-up of at least two years. All patients were operated on by the same surgeon.

In order to evaluate the level of chronic BCRL and to assess its regression after treatment, we decided to use circumference measurements of the arms as an objective clinical correlate. We measured the circumference of both the lymphedematous and the unaffected arm at six locations, namely at the level of the thumb saddle joint, the level of the wrist as well as the wrist plus 10 cm, 20 cm, 30 cm and 40 cm. All 12 measurements were taken preoperatively and then 6 weeks as well as 3, 6, 12 and 24 months postoperatively. Additionally, the stage of lymphedema was determined as well [37].

All complications that occurred during the follow-up period were recorded and classified according to Clavien-Dindo [38].

### Statistical analysis

We present a summary of the patients’ characteristics analyzed as mean and standard deviation (sd), median and interquartile ranges (iqr), as well as minimum and maximum value. We performed two-sample *t*-tests to compare means of continuous variables among patients that received VLNT + WAL and those that received VLNT alone. Chi square tests were used to examine difference in proportion for categorical variables. Statistical significance was determined at *p*-value < 0.05. All statistical analyses were performed using R version 4.0.1 (2020-06-06).

## Results

### Patient-specific variables

97 patients met the inclusion criteria: 53 (54.64%) patients underwent VLNT and 44 (45.36%) received a one-stage combination of VLNT with WAL. The median age was significantly higher in the VLNT + WAL group with 58.30 (SD = 10.00) years versus in 55.80 (SD = 7.90, *p* = 0.0130) years in the VLNT group. All patients were female. There was no significant difference in BMI between the two groups with a mean BMI of 28.60 (SD = 5.30) in the VLNT + WAL and 26.80 (SD = 3.90) in the VLNT group. Similarly, the handedness did not statistically differ with 42 (SD = 79.20) in the VLNT and 37 (84.10) in the VLNT + WAL group being right-handed and 3 (5.70) in the VLNT and 1 (2.30) in the VLNT + WAL group being left-handed (*p* = 0.6790).

### Regression of chronic lymphedema and surgery time

There was no significant difference in the clinical stage of chronic BCRL (*p* = 0.7770): 49 (SD = 92.50) patients in the VLNT and 39 (SD = 88.60) patients in the VLNT + WAL group had stage II chronic lymphedema. 2 (SD = 3.80) in the VLNT and 3 (6.80) in the VLNT + WAL group had stage III chronic lymphedema. For two patients in each group the stage was not recorded.

**Fig. 1:**
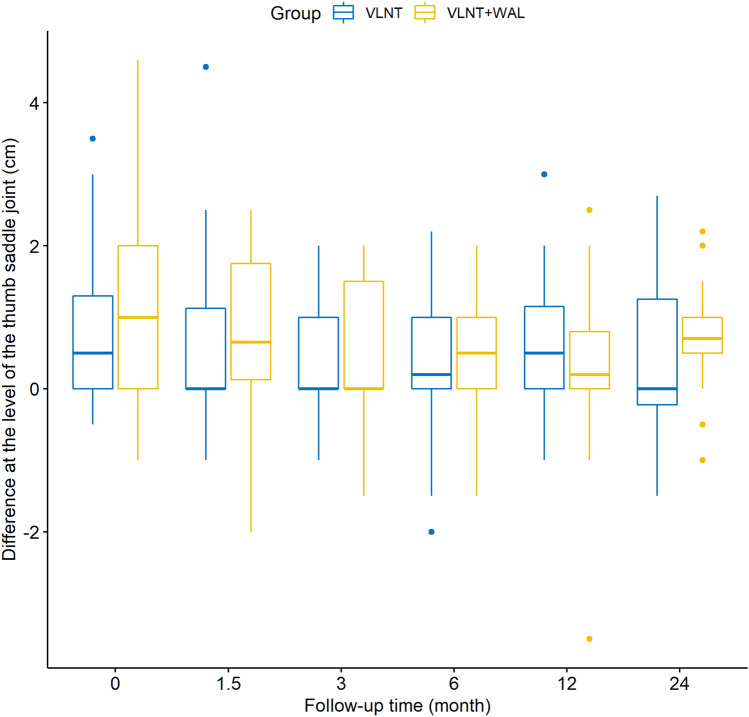
Differences of the circumferences between the lymphedematous and the unaffected arm at the level of the thumb saddle joint

**Fig. 2:**
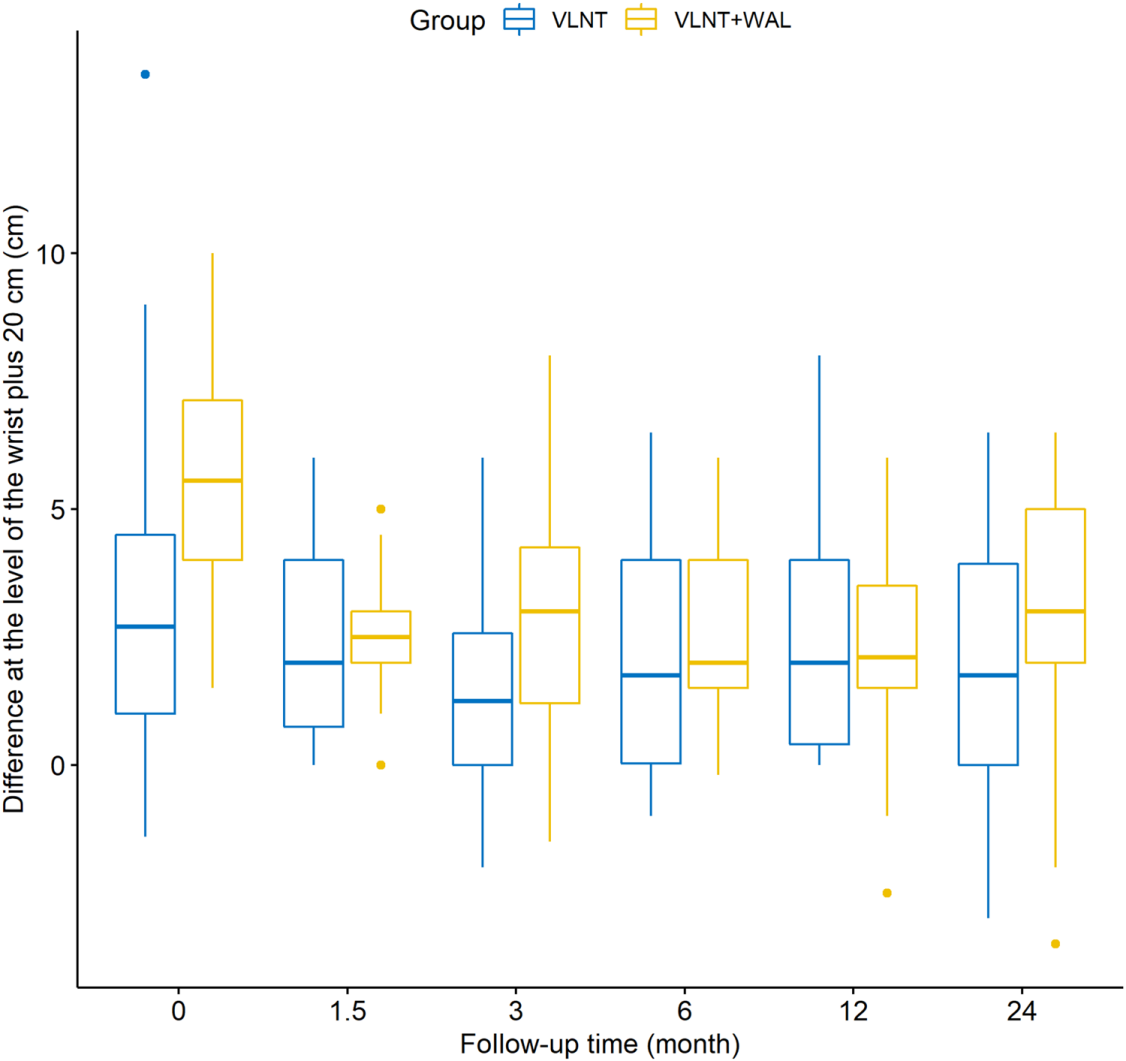
Differences of circumferences between the lymphedematous and the unaffected arm at the level of the wrist plus 20 cm

**Fig. 3:**
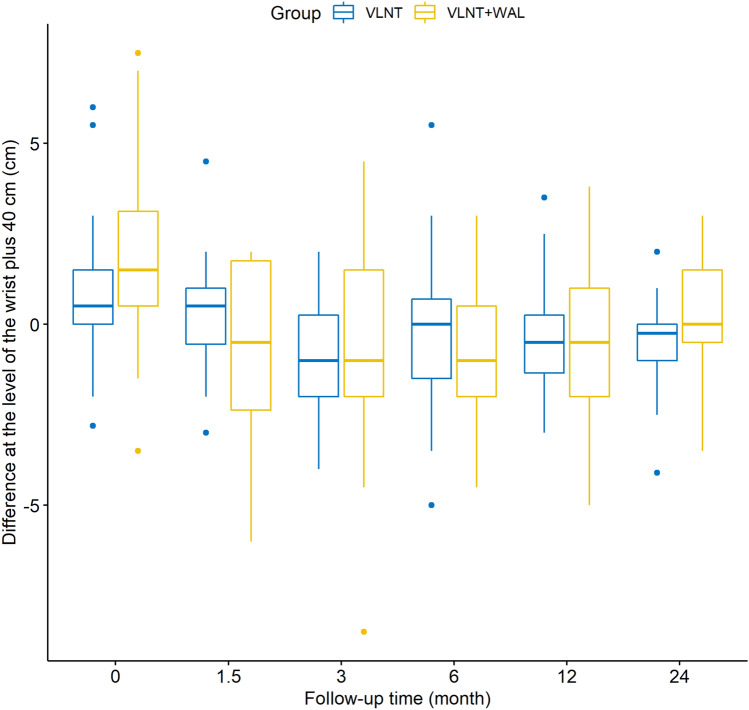
Differences of circumferences between the lymphedematous and the unaffected arm at the level of the wrist plus 40 cm

The mean circumferences at the level of the wrist, wrist plus 10, 20, 30 and 40 cm measured preoperatively on the lymphedematous arms differed significantly between our two groups (Table 1). With the exception of the circumferences measured at the level of the wrist, wrist plus 10 and 20 cm 24 months postoperatively, none of the mean circumferences of the lymphedematous arms measured postoperatively showed any significant differences between the two groups across the entire follow-up of 24 months (Table 1). With regard to the circumferences of the unaffected arms, we did not observe any significant differences between the VLNT and VLNT + WAL group—neither pre- nor postoperatively—with the exception of the circumferences measured at the level of the wrist plus 20 cm at 12 months follow-up (*p* = 0.0257). However, as for the differences of circumferences between the lymphedematous and the unaffected arm in an individual patient, the means were statistically different when measured preoperatively between the two groups (Figs. 1–3, Table 2). This holds true for measurements at all levels except at the level of the thumb saddle joint.

**Table 1:** Mean circumferences of the lymphedematous arms between groups: *p*-values

Location of measurement	Preoperative	1.5 months postoperative	3 months postoperative	6 months postoperative	12 months postoperative	24 months postoperative
Thumb saddle joint	0.190	0.437	0.467	0.306	0.919	0.562
Wrist	*0.008*	0.731	0.225	0.643	0.500	0.031
Wrist plus 10 cm	*0.00004*	0.669	0.092	0.395	0.086	0.018
Wrist plus 20 cm	*0.000004*	0.224	0.006	0.097	0.077	0.023
Wrist plus 30 cm	*0.00001*	0.400	0.594	0.913	0.574	0.072
Wrist plus 40 cm	*0.002*	0.441	0.503	0.779	0.738	0.396

The values in italic are values that are statistically significant (p < 0.05)

**Table 2:** Mean differences of circumferences of the lymphedematous and the unaffected arms between groups: *p*-values

Location of measurement	Preoperative	1.5 months postoperative	3 months postoperative	6 months postoperative	12 months postoperative	24 months postoperative
Thumb saddle joint	0.163	0.706	0.416	0.511	0.257	0.522
Wrist	*0.003*	0.907	0.121	0.929	0.562	0.37
Wrist plus 10 cm	*0.00001*	0.813	0.193	0.906	0.457	0.484
Wrist plus 20 cm	*0.000003*	0.632	*0.026*	0.644	0.919	0.360
Wrist plus 30 cm	*0.000003*	0.279	0.967	*0.019*	0.409	0.422
Wrist plus 40 cm	*0.002*	0.143	0.819	0.205	0.618	0.120

The values in italic are values that are statistically significant (p < 0.05)

The analysis revealed a significantly longer mean surgery time in the VLNT + WAL group than in the VLNT group (266.80 min, SD = 55.80 versus 195.60 min, SD = 39.60, respectively, *p*-value = 0.0003).

### Postoperative complications and patient satisfaction

There were no statistically significant differences for postoperative complications (Table 3): 5 (SD = 9.40) patients in the VLNT group developed a seroma (Clavien-Dindo grade I) postoperatively compared to 1 (SD = 2.30) patient in the VLNT + WAL group (*p* = 0.1070). Other complications included wound healing disorders at the recipient site (Clavien-Dindo grade IIIa) occurring in 2 (SD = 3.80) of the VLNT and in 1 (SD = 2.3) of the VLNT + WAL group (*p* = 0.2300) and hematomas (Clavien-Dindo grade I) occurring in 2 (SD = 3.80) patients in the VLNT and in 2 (SD = 2.30) patients in the WLNT + WAL group as well (*p* = 0.2280).

**Table 3:** Complications according to Clavien-Dindo

Clavien-Dindo	Complication	Therapeutic management		VLNT group	VLNT + WAL group	*p*-value
I	Seroma	Needle aspiration	Number (SD)	5 (9.40)	1 (2.30)	0.107
I	Hematoma	Needle aspiration/evacuation	Number (SD)	2 (3.80)	2 (4.50)	0.228
I	After Bleeding	Hemostasis through pressure dressing	Number (SD)	1 (1.90)	0	0.550
I	Convulsive attack	Nihil	Number (SD)	1 (1.90)	0	0.550
II	Erysipelas	Antibiotics	Number (SD)	1 (1.90)	0	0.550
IIIa	Prolonged secretion groin	Redon’s suction drainage	Number (SD)	0	1 (2.30)	0.550
IIIa	Wound healing disorder at recipient site	Dressing changes and vacuum-assisted closure therapy	Number (SD)	2 (3.80)	1 (2.30)	0.230
IIIa	Wound healing disorder at donor and recipient site	Dressing changes and vacuum-assisted closure therapy	Number (SD)	0	1 (2.30)	0.230
IIIb	Edge necrosis	Surgical debridement	Number (SD)	1 (1.90)	0	0.141
IIIb	Fat necrosis	Surgical debridement	Number (SD)	2 (3.80)	0	0.108
IIIb	Partial flap loss	Surgical revision	Number (SD)	1 (1.90)	0	0.141
IIIb	Total flap loss	Surgical revision	Number (SD)	1 (1.90)	0	0.141

On a numeric rating scale from zero to 10 with zero representing the highest and 10 the lowest level of satisfaction, no significant difference could be found for the level of patient satisfaction following VLNT or VLNT + WAL: the mean patient satisfaction in the VLNT group was 1.80 (SD = 0.80) versus 1.40 (SD = 0.70) in the VLNT + WAL group (*p* = 0.323).

## Discussion

### Combining VLNT with WAL

When evaluating the regression of chronic BCRL, the most objective and convenient variable is the difference of circumference between the lymphedematous and unaffected arm: by forming differences in each set of arms one takes into account the innate interindividual differences in arm circumference. This objective nature is akin to lymphedema severity which is based on increase in extremity volume in percentage and measured, for instance, via arm circumferences and categorized as mild, moderate or severe [37]. In contrast, lymphedema staging is based solely on clinical presentation, for example whether or not the lymphedema is pitting. [37, 39] In our study, we only recorded cases of lymphedema stage II and III and no significant differences between our two groups: lymphedema stage II refers to irreversible lymphedema while stage III includes fibroadipotic tissue and skin changes [37]. Therefore, lymphedema staging does not capture the whole picture—the objective severity—and simply represents another tool to assess chronic lymphedema. It is also somewhat imprecise as each stage of lymphedema has a large range of variation when looking at the objective increase in extremity volume.

This study gives further evidence that VLNT leads to favourable patient outcomes as multiple studies and systematic reviews have demonstrated [22, 27]. In all 53 patients who underwent VLNT alone, the mean differences of circumferences between the lymphedematous and unaffected arm decreased postoperatively over the follow-up period of 24 months (Figs. 1, 2, 3, Table 2).

Our results show that the mean differences of circumferences of the lymphedematous and unaffected arms measured preoperatively in each individual patient was significantly higher in the VLNT + WAL than in the VLNT group (excluding the differences of circumferences measured at the level of the thumb saddle joint). Logically, this means that the extent of chronic BCRL as expressed by increase in extremity volume in the VLNT + WAL group was greater than in the VLNT group. Yet, for the majority of measurements, the differences of circumferences between the lymphedematous and unaffected arm measured postoperatively in each individual patient were not significantly higher in the VLNT + WAL than in the VLNT group. It follows that because WAL was used to treat patients with larger circumferences of the lymphedematous arm, these patients attained outcomes comparable to the patients who initially had milder chronic BCRL and thus were not also treated with WAL. So even though these patients treated additionally with WAL had worse chronic BCRL they still attained excellent results not differing significantly from those achieved in the patients with milder chronic BCRL which clearly speaks to the merits of a one-stage combination of VLNT with WAL. It stands to reason that the correlation between greater difference of circumference between lymphedematous and unaffected arm in an individual patient and thus more severe chronic lymphedema and a surgeon selecting to combine VLNT with WAL in a one-stage procedure is founded on clinical experience. Lastly, if severer cases of chronic BCRL can be adequately treated in a one-stage operation—as was the case for our patients in the VLNT + WAL group—oftentimes necessary secondary interventions can be avoided which is certainly in the interest of the patient.

Unsurprisingly, the mean surgery time was longer in the VLNT + WAL group. This point might generally be relevant as there is a correlation between operating time and surgical complications [40]. Nevertheless, there was no higher occurrence of postoperative complications in the VLNT + WAL group. Also, each minute in the operating room is tied to considerable costs [41]. However, this last point seems especially moot in light of our results given that a second intervention and thus increased patient burden is avoided and since an improved treatment of chronic BCRL will doubtlessly save costs over time.

### Complications

Our results show that complications following VLNT or a one-stage combination of VLNT with WAL are seldom. This is in line with a systematic review by Scaglioni et al. who found relatively low complication rates after VLNT [42]. Furthermore, complications associated with liposuction in general, e.g. wound infection, can be effectively prevented [43]. In fact, we found no liposuction associated complications. Despite the fact that adrenaline is used in tumescent solution in WAL which causes a vasoconstriction of blood vessels within the flap and the increased surgery time in the VLNT + WAL group, we uncovered no significant difference in complications between our two groups.

### Personalized medicine

The paradigm of one size fits all will shift to a highly personalized form of practicing medicine in the future [44, 45]. This will not just affect drug therapies but also the field of surgery resulting in enhanced patient outcomes [46]. As our results would suggest, it is not always necessary to use liposuction concurrently with VLNT to treat chronic BCRL. Accordingly, for patients with milder forms of chronic BCRL, VLNT alone would appear to suffice in order to achieve a significant reduction in morbidity. Conversely, our results suggest that patients with severe chronic BCRL seem to benefit from treatment with a one-stage combination of VLNT with WAL.

### Study strengths and limitations

The main strength of our study is the relatively large sample for a study in the field of (lymphatic) surgery. To our knowledge, it is the largest for any study comparing VLNT with WLNT + WAL to date [31, 32]. This allows for a more exact estimate of treatment effect and increases generalizability of our results. Moreover, our study included meticulous circumference measurements of the lymphedematous and unaffected arm pre- and postoperatively. Hence, we were able to form differences in individual sets of arms taking into account the interindividual anatomical variation. The study by Leppäpuska et al. which also examined the combination of VLNT with WAL, however, did not take such measurements of both the lymphedematous and unaffected arm pre- and postoperatively due to which differences could not be formed [32]. A main limitation of our study is its retrospective design which resulted in patients with more severe chronic BCRL being in the VLNT + WAL group. This unequal distribution of patients could be prevented by a prospective and randomized study design. The absence of validated patient-reported outcome measures (PROMs) represents another study limitation. Seeing as BCRL can significantly impact quality of life, the use of validated PROMs such as the LYMPH-ICF or LYMPH-QOL in routine clinical practice and in future studies would be all the more important as they reflect the patient’s perspective on a given outcome [47, 48]. Although we consider validated PROMs to be highly important in the treatment of chronic BCRL, we were not able to use them in our study as they have only recently begun to gain traction in clinical practice. In light of these circumstances, a pragmatic, randomized and multicenter study would be appropriate with inclusion of validated PROMs like the LYMPH-ICF or the LYMPH-QOL.[48]

## Conclusions

Our data suggest that a one-stage combination of VLNT with WAL can be safely and effectively performed and results in improved patient outcomes as expressed by an increased reduction in arm circumference compared to VLNT alone. Validated PROMs ought to be implemented in routine clinical practice at all major treatment centers.

## Data Availability

The data which were collected, entered into our database and analyzed as part of this study is not publicly available in compliance with the ethics committee approval and due to their sensitive nature. Nevertheless, the data can be obtained from the corresponding author on reasonable request.

## Abbreviations

BCRL: Breast cancer-related lymphedema
VLNT: Vascularized lymph node transfer
WAL: Water jet-assisted liposuction

## References

[1] DiSipio T (2013). Incidence of unilateral arm lymphoedema after breast cancer: a systematic review and meta-analysis. Lancet Oncol.

[2] Maughan KL, Lutterbie MA, Ham PS (2010). Treatment of breast cancer. Am Fam Physician.

[3] Özkurt E, Golshan M (2019). ASO author reflections: de-escalation of breast cancer surgery—a leap forward. Ann Surg Oncol.

[4] Morrow M (2017). De-escalating and escalating surgery in the management of early breast cancer. Breast.

[5] Jatoi I, Benson JR, Toi M (2016). De-escalation of axillary surgery in early breast cancer. Lancet Oncol.

[6] Bojinović-Rodić D (2016). Upper extremity function and quality of life in patients with breast cancer related lymphedema. Vojnosanit Pregl.

[7] Vassard D (2010). Psychological consequences of lymphoedema associated with breast cancer: a prospective cohort study. Eur J Cancer.

[8] Cidón EU, Perea C, López-Lara F (2011). Life after breast cancer: dealing with lymphoedema. Clin Med Insights Oncol.

[9] Taghian NR (2014). Lymphedema following breast cancer treatment and impact on quality of life: a review. Crit Rev Oncol Hematol.

[10] Smoot B (2010). Upper extremity impairments in women with or without lymphedema following breast cancer treatment. J Cancer Surviv.

[11] Ridner SH (2012). Breast cancer survivors with lymphedema: glimpses of their daily lives. Clin J Oncol Nurs.

[12] Ridner SH (2012). Voices from the shadows: living with lymphedema. Cancer Nurs.

[13] Khan F (2012). Factors associated with long-term functional outcomes and psychological sequelae in women after breast cancer. Breast.

[14] O’Toole J (2013). Lymphedema following treatment for breast cancer: a new approach to an old problem. Crit Rev Oncol Hematol.

[15] Ridner SH (2005). Quality of life and a symptom cluster associated with breast cancer treatment-related lymphedema. Support Care Cancer.

[16] Gutknecht M (2017). Cost-of-illness of patients with lymphoedema. J Eur Acad Dermatol Venereol.

[17] Dean LT (2019). “It still affects our economic situation”: long-term economic burden of breast cancer and lymphedema. Support Care Cancer.

[18] Boyages J (2016). Worse and worse off: the impact of lymphedema on work and career after breast cancer. Springerplus.

[19] Oremus M (2012). Systematic review: conservative treatments for secondary lymphedema. BMC Cancer.

[20] Heinig B, Wollina U (2015). Complex decongestive therapy. Hautarzt.

[21] Jeffs E (2018). Clinical effectiveness of decongestive treatments on excess arm volume and patient-centered outcomes in women with early breast cancer-related arm lymphedema: a systematic review. JBI Database System Rev Implement Rep.

[22] Ozturk CN (2016). Free vascularized lymph node transfer for treatment of lymphedema: a systematic evidence based review. J Plast Reconstr Aesthet Surg.

[23] Ngo QD (2020). Vascularized lymph node transfer for patients with breast cancer-related lymphedema can potentially reduce the burden of ongoing conservative management. Lymphat Res Biol.

[24] Park KE (2020). Surgical management of lymphedema: a review of current literature. Gland Surg.

[25] Schaverien MV (2018). Vascularized lymph node transfer for lymphedema. Semin Plast Surg.

[26] Ito R (2016). Proposed pathway and mechanism of vascularized lymph node flaps. Gynecol Oncol.

[27] Coriddi M (2020). systematic review of patient-reported outcomes following surgical treatment of lymphedema. Cancers (Basel).

[28] Schaverien MV, Munnoch DA, Brorson H (2018). Liposuction treatment of lymphedema. Semin Plast Surg.

[29] Forte AJ (2019). Lipoaspiration and controlled compressive therapy in lymphedema of the upper extremity: a comprehensive systematic review. Cureus.

[30] Forte AJ (2019). Lipoaspiration for the treatment of lower limb lymphedema: a comprehensive systematic review. Cureus.

[31] Forte AJ (2019). Lipoaspiration and lymph node transfer for treatment of breast cancer-related lymphedema: a systematic review. Cureus.

[32] Leppäpuska IM (2019). Combined surgical treatment for chronic upper extremity lymphedema patients: simultaneous lymph node transfer and liposuction. Ann Plast Surg.

[33] Dayan JH, Dayan E, Smith ML (2015). Reverse lymphatic mapping: a new technique for maximizing safety in vascularized lymph node transfer. Plast Reconstr Surg.

[34] Viitanen TP (2012). Donor-site lymphatic function after microvascular lymph node transfer. Plast Reconstr Surg.

[35] Zhang H (2014). The distribution of lymph nodes and their nutrient vessels in the groin region: an anatomic study for design of the lymph node flap. Microsurgery.

[36] Moffatt CJ (2003). Lymphoedema: an underestimated health problem. QJM.

[37] The diagnosis and treatment of peripheral lymphedema (2013). 2013 Consensus Document of the International Society of Lymphology. Lymphology.

[38] Clavien PA (2009). The clavien-dindo classification of surgical complications: five-year experience. Ann Surg.

[39] Greene AK, Goss JA (2018). Diagnosis and staging of lymphedema. Semin Plast Surg.

[40] Hardy KL (2014). The impact of operative time on complications after plastic surgery: a multivariate regression analysis of 1753 cases. Aesthet Surg J.

[41] Childers CP, Maggard-Gibbons M (2018). Understanding costs of care in the operating room. JAMA Surg.

[42] Scaglioni MF (2018). Comprehensive review of vascularized lymph node transfers for lymphedema: outcomes and complications. Microsurgery.

[43] Dixit VV, Wagh MS (2013). Unfavourable outcomes of liposuction and their management. Indian J Plast Surg.

[44] Goetz LH, Schork NJ (2018). Personalized medicine: motivation, challenges, and progress. Fertil Steril.

[45] Di Sanzo M (2017). Clinical applications of personalized medicine: a new paradigm and challenge. Curr Pharm Biotechnol.

[46] Harris EP (2019). Personalized perioperative medicine: a scoping review of personalized assessment and communication of risk before surgery. Can J Anaesth.

[47] Meadows KA (2011). Patient-reported outcome measures: an overview. Br J Community Nurs.

[48] Devoogdt N (2011). Lymphoedema functioning, disability and health questionnaire (Lymph-ICF): reliability and validity. Phys Ther.

